# “*It’s just a perfect storm”*: Exploring the consequences of the COVID-19 pandemic on overdose risk in British Columbia from the perspectives of people who use substances

**DOI:** 10.1186/s12889-023-15474-5

**Published:** 2023-04-03

**Authors:** Annie Foreman-Mackey, Jessica Xavier, Jenny Corser, Mathew Fleury, Kurt Lock, Amiti Mehta, Jessica Lamb, Jenny McDougall, Cheri Newman, Jane A. Buxton

**Affiliations:** 1 British Columbia Centre for Disease Control, 655 West 12th Avenue, Vancouver, BC V5Z 4R4 Canada; 2 Faculty of Medicine, University of British Columbia, 317 – 2194 Health Sciences Mall, Vancouver, BC V6T 1Z3 Canada; 3 Faculty of Health Sciences, SFU, Blusson Hall, Room 11300, 8888 University Drive, Burnaby, BC V5A 1S6 Canada; 4 First Nations Health Authority, 540-757 West Hastings St, Vancouver, BC V6C 1A1 Canada; 5 Professionals for Ethical Engagement of Peers, Peer Engagement and Evaluation Project, British Columbia Centre for Disease Control, 655 West 12th Avenue, Vancouver, BC V5Z 4R4 Canada; 6 School of Population and Public Health, University of British Columbia, 2206 East Mall, Vancouver, BC V6T 1Z3 Canada

**Keywords:** Overdose, People who use substances, COVID-19, Qualitative

## Abstract

**Background:**

Despite the implementation and expansion of public health and harm reduction strategies aimed at preventing and reversing overdoses, rates of overdose-related events and fatalities continue to rise in British Columbia. The COVID-19 pandemic created a second, concurrent public health emergency that further exacerbated the illicit drug toxicity crisis, reinforced existing social inequities and vulnerabilities, and highlighted the precariousness of systems in place that are meant to protect the health of communities. By exploring the perspectives of people with recent experience of illicit substance use, this study sought to characterize how the COVID-19 pandemic and associated public health measures influenced risk and protective factors related to unintentional overdose by altering the environment in which people live and use substances, influencing the ability of people who use substances to be safe and well.

**Methods:**

One-on-one semi-structured interviews were conducted by phone or in-person with people who use illicit substances (n = 62) across the province. Thematic analysis was performed to identify factors shaping the overdose risk environment.

**Results:**

Participants pointed to factors that increased risk of overdose, including: [1] physical distancing measures that created social and physical isolation and led to more substance use alone without bystanders nearby able to respond in the event of an emergency; [2] early drug price spikes and supply chain issues that created inconsistencies in drug availability; [3] increasing toxicity and impurities in unregulated substances; [4] restriction of harm reduction services and supply distribution sites; and [5] additional burden placed on peer workers on the frontlines of the illicit drug toxicity crisis. Despite these challenges, participants highlighted factors that protected against overdose and substance-related harm, including the emergence of new programs, the resiliency of communities of people who use substances who expanded their outreach efforts, the existence of established social relationships, and the ways that individuals consistently prioritized overdose response over concerns about COVID-19 transmission to care for one another.

**Conclusions:**

The findings from this study illustrate the complex contextual factors that shape overdose risk and highlight the importance of ensuring that the needs of people who use substances are addressed in future public health emergency responses.

## Background

Responding to a rise in fatal drug overdoses, British Columbia (BC) declared a public health emergency under the *Public Health Act* in 2016 resulting in the expansion of public health and harm reduction strategies aimed at preventing and reversing overdoses [1]. Although the overall rates of overdose have largely continued to increase following the declaration, there was a marked reduction in the lives lost in 2019, from 31.1 deaths per 100,000 in 2018 to 19.3 deaths per 100,000 in 2019 [2]. Illicit fentanyl and its analogues in the unregulated drug market is driving the increase in overdose events and deaths in BC; fentanyl has been detected in more than 80% of illicit drug toxicity deaths in BC since 2017, a dramatic increase from 5% to 2012 and 25% in 2014 [3]. The onset of the COVID-19 pandemic in 2020 brought about a second, concurrent public health emergency that – along with the associated public health measures enacted to reduce the spread of the virus, such as physical distancing – led to an increased risk of overdose and drug-related harm [4].

Since the COVID-19 pandemic was declared, the unregulated drug supply has become even more toxic with post mortem toxicology results indicating a greater number of cases with extreme fentanyl concentrations (> 50 micrograms per litre) identified in decedents increasing from 8% between January 2019 to March 2020 all the way to 13% and 16% in April 2020 to October 2021 and November 2021 to August 2022 respectively [3]. In addition, the detection of benzodiazepine-like substances increased rapidly from 15% in July 2020 to 52% of samples in January 2022 [3]. Benzodiazepine contaminants in unregulated opioids further increase the risk of overdose, causing prolonged sedation, dependence, and benzodiazepine withdrawal when individuals are no longer able to access illicit opioids. The BC Coroners data also indicates that 83% of illicit drug toxicity deaths occurred in private or other residences [2]. This context undermined the early progress made in BC with respect to harm reduction program expansion and reductions in fatalities, and triggered the largest yearly increase in drug toxicity deaths since 2010 [5]. Illicit drug toxicity deaths surpassed historic highs during the pandemic, with 2,267 people dying from overdose in 2021, up from 1,775 to 2020 and 984 in 2019; and deaths in 2022 will be similar to 2021 [2].

The COVID-19 pandemic has highlighted existing social inequities and vulnerabilities, as well as the precariousness of systems in place to protect the health of communities [6, 7]. Many people who use substances are particularly vulnerable to COVID-19 given high rates of pre-existing chronic health conditions, ongoing stigma and discrimination, and other social vulnerabilities among marginalized people who use drugs such as inadequate housing and poor access to health services [7–11]. Public health measures such as physical distancing, service delivery restrictions and closures were put in place to curb transmission of the virus. However, as noted in previous literature, these have the potential to disproportionately impact vulnerable groups and run the risk of further entrenching social inequities and harm among people who use substances [6, 9, 13]. Notably, these types of interventions are believed to increase risk of overdose by creating a more toxic and inconsistent supply of illicit substances [13–15], decreasing access to harm reduction and other health and social services [6, 8, 16, 17], and causing people to more frequently use drugs alone without other individuals nearby and able to intervene in the event of an overdose [12, 14, 18, 19].

The ‘risk environment’ framework identifies complex interactions between individuals and their broader environments as key determinants of individual risk and protective factors as well as distribution of harm among populations [20–22]. It is often applied throughout harm reduction, drug policy, and substance use research to characterize the impact of structural factors on health outcomes and behaviours [19, 22]. The framework outlines four key risk environment domains – physical (e.g. drug use settings), social (e.g. group norms), economic (e.g. drug prices), and policy (e.g. drug criminalization) – that are external to the individual but shape individual risk and behaviours [22]. This paper draws on elements of the risk environment framework to interpret and discuss our findings and the ways that the COVID-19 pandemic and emergent responses have altered the environments in which people live and use substances, thereby producing additional risk of overdose and other substance use related harms. Research to date has examined current trends and postulated causative pathways for the drastic rise in unintentional overdose fatalities [5], however, the extent of the impact of the pandemic on people who use substances has yet to be fully determined [23] and few studies have directly explored the experiences of people who use substances in the midst of the COVID-19 pandemic [17, 24–27].

By exploring the perspectives of people with lived and living experience of substance use in BC, we sought to characterize how the COVID-19 pandemic and associated public health measures influenced protective factors and risks related to unintentional overdose by altering the environment in which people live and use substances.

## Methods

This study consisted of semi-structured, one-on-one interviews conducted by phone or in-person as part of two qualitative studies performed across BC: the Concurrent Use and Transition to Methamphetamine among People at Risk of Overdose (CUT Meth OD) study (n = 27) and the Good Samaritan Drug Overdose Act (GSDOA) evaluation study (n = 35) [28, 29]. Qualitative interview guides were developed by the research team – including researchers, people with lived and living experience of substance use, stakeholders from community organizations, health authorities, and young adults. Interview guides included questions that explored the impact of COVID-19 on: access to and availability of substances, harm reduction services, and supplies; ability to “buddy up” (having someone close by to help in the event of an emergency); and concerns about COVID-19 when responding to an overdose and the potential impacts on bystanders’ response to overdoses.

Qualitative interviews were conducted between October 2020 and May 2021 by academic researchers and peer research assistants (PRAs) from across the province, some of whom were enrolled from the Professionals for Ethical Engagement of Peers (PEEP), a peer advisory group at the BC Centre for Disease Control. PRAs completed the Tri-Council Policy Statement-2 tutorial on Ethical Conduct for Research Involving Humans and received research and interview training, including practice interviews in advance of data collection.

Participants were recruited through referral from harm reduction sites, youth organizations, word of mouth, and snowball sampling. Participants were included if they provided informed consent, were able to speak and understand English, were 16 years of age or older, and self-identified as using illicit substances currently or within the past month. Interviews lasted between 30 and 90 min and were audio-recorded, transcribed verbatim, and de-identified to ensure participant confidentiality and anonymity. Participants completed a brief, anonymous questionnaire at the start of each interview to collect demographic and substance use details. Participants in the GSDOA study received a $20 honorarium and CUT Meth OD study participants received a $30 honorarium for a longer interview.

Our aim was to explore experiences of people who use substances during the COVID-19 pandemic by combining inductive and deductive approaches. Interview transcripts were imported into NVivo Qualitative Data Analysis Software to organize the data. A descriptive code – ‘COVID-19’ – identified all text relevant to participants’ experiences and perspectives of COVID-19. Data under the COVID-19 code were exported from NVivo into a Microsoft Word document for analysis. These data included answers to specific COVID-19 interview questions but also where COVID-19 was mentioned anywhere during the interviews.

We adopted an analytic approach that draws on elements of applied thematic analysis for this paper given a need to both explore and interpret rich and detailed data [30, 31]. Preliminary analysis was conducted by the lead author (AFM) with input from the research team throughout the analysis process. AFM reviewed the transcript excerpts to become familiar with the data. AFM generated initial codes using open-ended coding to sort the data and build a preliminary coding framework which was discussed with study team members (JB, JX, AM, JC, MF). Emerging themes were identified and a mind map was developed to graphically explore the interconnected nature of the themes. The data were further explored to capture additional themes or emerging subthemes.

Preliminary findings were reviewed by co-authors and members of PEEP who provided input related to the analysis and interpretation of the data, as well as the presentation of findings to ensure they were non-stigmatizing and aligned with the experiences of people and communities who use substances. The ‘risk environment’ framework [20, 21] was used to provide context for the interpretation of the findings and enhance the discussion.

Simple descriptive statistics of participant characteristics were computed using Microsoft Excel. Both studies included in this paper were approved by the University of British Columbia’s research ethics board.

## Results

Table 1 outlines the demographic and substance use characteristics of the 62 participants. Participants were diverse with respect to age, location of residence, gender, and indigeneity and most participants reported using both opioids and stimulants.

**Table 1:** Summary characteristics of the study population

Participant Characteristics	Number of Participants (%) N = 62
***Age***	
18–24	11 (17.7%)
25–34	14 (22.6%)
35–44	16 (25.8%)
45–54	10 (16.1%)
55+	8 (12.9)
Unknown ^a^	3 (4.8%)
***Urbanicity*** ^***b***^	
Rural	2 (3.2%)
Small Urban	8 (12.9%)
Medium Urban	3 (4.8%)
Large Urban	29 (46.8%)
Metropolitan	20 (32.3%)
***Gender Identity***	
Woman	26 (41.9%)
Man	29 (46.8%)
Gender non-conforming, intersex, non-binary or transgender	4 (6.5%)
Unknown ^a^	3 (4.8%)
***Indigeneity*** ^***c***^	
Yes	29 (46.8%)
No	30 (48.4%)
Unknown ^a^	3 (4.8%)
***Opioid Use***	
Yes	40 (64.5%)
No	15 (24.2%)
Unknown ^a^	7 (11.3%)
***Stimulant Use***	
Yes	50 (80.6%)
No	5 (8.1%)
Unknown ^a^	7 (11.3%)
***Use both Stimulants and Opioids***	
Yes	35 (56.5%)
No	20 (32.3%)
Unknown ^a^	7 (11.3%)

^*a*^ *Unknown represents data that was not reported by participants.*

^*b*^*Urbanicity of participant locations of residence were classified according to a system developed by the BC Ministry of Health which relies on definitions created by Statistics Canada* [32], *while also taking into consideration remoteness, population density, and proximity to urban areas* [33].

^*c*^*Indigeneity self-reported by participants as Yes/No in the GSDOA Study and included participants self-identifying as First Nations and Métis in the CUT Meth Study. It is important to note that Indigenous identity often serves as a proxy for the downstream impacts of colonialism, including but not limited to socioeconomic status, intergenerational trauma, and the systemic racism experienced by Indigenous individuals* [34].

### Factors shaping risk of overdose and substance use-related Harms

We identified five overarching themes shaping overdose risk and substance use-related harms as a consequence of the COVID-19 pandemic: [1] physical distancing measures created social and physical isolation which in turn led to increased substance use and more often using alone without bystanders nearby able to respond in the event of an emergency; [2] inconsistent drug availability and quality due to supply chain issues with early drug price spikes and increasing toxicity and impurities in unregulated substances; [3] stigma towards people who use substances which was compounded by COVID-19; [4] access to harm reduction services and supply distribution sites were restricted in some locations early in the pandemic; and [5] additional burden was placed on peer workers on the frontlines of the illicit drug toxicity crisis. These factors and associated findings are presented in the following sections and later presented within the context of the ‘risk environment’ framework.

#### Physical distancing, increased drug use and using drugs alone

For many participants, social and physical isolation brought about by the pandemic and physical distancing restrictions eroded their mental health and led to increased drug consumption. The pandemic also brought about a loss of readily available jobs and economic opportunities, which meant that people were at home for extended periods of time and struggling financially “*to make ends meet”* (#6, large urban). Participants explained that the challenges brought on by the COVID-19 pandemic of increased drug consumption, using alone and drug toxicity contributed to a striking increase in overdoses: “*the COVID context… with the drug toxicity… it’s just a perfect storm and it’s just a nightmare*” (#53, large urban).

One participant noted that substance use “*went from ten to a thousand from what I’ve seen… Everywhere you go you see people you don’t even think of doing it, is doing it”* (#6, large urban). Another participant noted that before the pandemic, they were using substances “*three days every fucking couple weeks or whatever.* [However, during the pandemic] *I’ve been doing it every day… this whole past year… It’s ‘cause of COVID-19, I think*” (#22, small urban).

Young adult participants aged 18–24 described the additional difficulties in managing their mental health in the context of the pandemic, especially while isolating in challenging family settings and with limited access to their usual social networks and supports, leading to increased substance use for some during this period of time.

[COVID-19] *has made it a lot harder to go out. My anxiety and depression have obviously increased. I find it’s a lot harder to get through some things when you can’t, like, go and visit a friend per se or see your youth worker in person.* (#55, metropolitan)

Many participants felt that physical distancing restrictions led to more individuals consuming drugs in private locations, separate from peers and bystanders able to intervene in the event of an emergency. They identified these factors as critical drivers of the rising rates of overdose fatalities.

*What scares me about people using alone, is just the safety risk to me. You can’t – if you OD you can’t call 911 on yourself. It’s just that higher rate for fatality if there’s an overdose… And if I had to guess why more people are dying from overdosing rather than overdosing and being brought back, it’s because they’re using alone.* (#54, large urban)

In addition, some supportive housing sites also implemented stricter visitor policies in the context of the COVID-19 pandemic: “*a lot of people in those buildings aren’t allowed to have people in there with them*” (#46, large urban). This may have increased people using alone and reduced the ability for ‘spotting’ or peer witnessing, where individuals observe others using substances and are present to respond if an overdose occurs.

However, not all participants perceived distancing guidelines as having an impact on substance use patterns. Several participants indicated that physical distancing “*goes out the window when you’re talking about drugs*” (#17, metropolitan). Another participant felt that they were still able to buddy up when using drugs: “*We usually don’t maintain social distancing to be honest… some people help others to inject… or they share things… To trade goods and stuff you have to be within a certain distance of someone. So it’s really hard*” (#25, medium urban). As such, the inherently social nature of drug use acquisition and use for many people meant that physical distancing was not a factor in how they acquired, prepared, and used their drugs.

While some participants expressed fear of contracting COVID-19, leading them to remain indoors alone or more closely follow COVID-19 physical distancing orders, others said that they were not concerned about being infected with the virus: “*We live in a world of our own, you know. I said that if I was going to go, like, go, I would have already… fentanyl would have already taken me. So if it’s going to be COVID, then that’s silly*” (#6, large urban). For these participants, individual risk of overdose outweighed concerns about COVID-19. In addition, as some suggested, individual perception and tolerance of risk among people who use substances may differ from other segments of the population given frequent exposure to lethal substances and potential risk of harm in their daily lives. The perception of COVID-19 risk among these participants was in direct contrast to other participants who described feeling a sense of enhanced vulnerability to the virus given high rates of co-morbid conditions among people who use substances.

These findings demonstrate that people who use substances represent a heterogeneous group with variable perceptions of and exposures to risk, health conditions and contextual realities (e.g. economic, social, demographic). These factors contribute to the unique impact of COVID-19 and associated public health measures on certain communities and one’s ability to follow suggested measures. People who use substances often had to weight relative risks in the context of dual pandemics to maintain their safety.

#### Drug availability and quality

Many participants noted that the price of substances sold on the street spiked significantly early in the pandemic before stabilizing in the following months.

*It got incredibly expensive and then dropped in price again… right around the start of the pandemic… the price started rising incredibly. Like, it tripled in price almost at one point. And then – yeah, and then it’s since come down, back to basically its normal price.* (#17, metropolitan)

The rising prices had disproportionate impact on certain communities, impacting their ability to access their drug of choice: “*150 bucks, 200 bucks more isn’t much money* [for higher income individuals]. *But on the level of the street people it is… It’s the access to what’s getting to the lower population is garbage. But people with money, they get the good shit. It’s the way it is. It’s gross*” (#23, metropolitan). This participant noted that people who use substances coming from lower socioeconomic backgrounds not only faced financial barriers in accessing substances, but also had limited ability to be selective, forcing many to turn to a more affordable, but also more unreliable, toxic supply. Rural communities faced additional challenges in accessing substances. Participants explained that drug prices in rural areas of BC remained elevated for longer and that the supply chain disruptions meant that substances were being increasingly cut with other substances by the time they arrived in their jurisdictions.

Participants spoke of inconsistencies in the drug supply and more frequent “dry spells” that disrupted their usual patterns of use. Participants pointed to dry spells as a reason for changing their regular drug of choice to whatever is available: “*you move on to what’s available*” (#57, metropolitan) and “*people were getting desperate. And I think they were doing whatever they had to do, right, ‘cause you couldn’t get it”* (#26, small urban).

Participants also pointed to the safety and comfort they felt when acquiring substances from a trusted supplier. The shifting drug availability disrupted this sense of trust, when suppliers were unable to provide their usual substances: “*the people that I trust with my safe supply [dealers with a trusted supply of illegal drugs] no longer have that safe supply”* (#58, metropolitan). Some individuals coped by either turning to new or unknown suppliers, or by adjusting their substance use to match what was available from their established source of unregulated supply.

*Someone who might usually sell pure cocaine that’s made by a trusted person who makes drugs is now, like, we, you know I can’t get my hands on that and they stopped making it ‘cause they can’t get their hands on that. So I actually sell meth now. So if you want drugs your options are meth or nothing. And a lot of drug users don’t have a choice right now. Like, well, I need something so I’m going to have to go with meth.* (#54, large urban)

Despite the rising prices and inconsistencies in availability, some participants felt that they were still able to access substances when desired by relying on social capital: “*I know a lot of people and stuff so it’s still the same amount, you know, if I can’t get it at one place I go to another. I usually seem to always get it”* (#16, large urban). Similarly, individuals spoke of sharing substances when they faced an inconsistent supply of substances: “*A lot of people look out for each other out here. And so, like, if other people have* [substances] *they’re more than willing to give it to you.*” (#6, large urban). Participants felt that this collectivist mentality and commitment to keeping each other well and safe, served to protect them from additional harms brought about by dry spells and inconsistent drug availability. However, this may not be possible for those without social capital or lacking networks of relationships.

In addition to facing an inconsistent supply of substances, participants also explained that the available drugs were becoming increasingly contaminated and dangerous. For example, participants said that: *“Cocaine right now is not cocaine. It’s straight up hog dewormer* [levamisole]*”* (#58, metropolitan) and *“it’s basically stripper. So you’re drinking paint stripper”* (#23, metropolitan). Concerns about contamination extended beyond opioids and fentanyl to include stimulants.

Participants felt that they were at a heightened risk of overdose and harm due to the toxicity and unpredictability of the drug supply. One participant explained that: “*You’re used to doing two points so – even being safe they think doing a point is safe in comparison and actually it’s not*” (#12, small urban). Participants described feeling fearful given uncertainty about what substances and contaminants were in the street drugs they were consuming which undermined their safety norms.

*I see a lot of fear in people who use now… There’s a lot of people going down. There’s a lot of fentanyl everywhere, especially being mixed in with benzos. It’s, like, kind of the worst… people can’t use alone anymore. That’s not even an option ‘cause it’s, like, so highly probable that you’re going to end up dead if you do use alone*. (#58, metropolitan)

Overall, participants felt that the effects of both the increasingly toxic unregulated drug supply alongside issues of access due to price increases and supply chain inconsistencies served to increase risk of overdose and drug-related harm throughout the early pandemic. Participants shared that while having a trusted source could be protective against an increasingly toxic drug supply, the pandemic reduced and removed opportunities to be selective when purchasing substances given supply chain issues at all levels, including low-level dealers’ supply.

#### Stigma compounded by COVID-19

Participants identified ongoing stigma towards people who use substances and felt that it was intensified and exacerbated by additional judgment and perception of marginalized and precariously housed people who use substances as “dirty” and therefore more “infectious” in the context of COVID-19. Some participants explained that they attempt to keep their substance use hidden because “*there is stigma and judgment towards people that are… out here on the street or using drugs… I think they have their own preconceived judgment towards or their idea of… what drug addicts are. As far as they know they’re dirty… it is pretty scary*” (#37, metropolitan). Participants felt that the pandemic led to a snowball of harmful associations between individuals who use substances and the tendency to contract and transmit COVID-19.

*A lot of people are afraid of the downtown community, downtown core. They think that it’s infectious down here and that you have a much higher risk of getting COVID down here. Which I don’t believe. There’s the buses and stuff, the systems, the airplanes, all places like that and I’m not really sure of. But I’d say you have just about as much risk of catching it there as you do down here.* (#25, medium urban)

Other participants expressed that they felt that during the COVID-19 pandemic, the needs of people who use substances who were impacted by the illicit drug toxicity crisis were overlooked: “*the public don’t give a shit about* [people who use substances]” (#28, large urban), and “*some organizations have definitely turned their back on* [people who use substances] *and it’s discouraging to see*” (#10, large urban), as seen by more restricted access to and closure of some harm reduction services. This perceived of lack of support and increased discrimination as well as the public fear of the downtown community being more likely to have and spread COVID-19 made participants feel even more stigmatized and marginalized.

#### Access to services and supplies

Some participants indicated that access to harm reduction services and supply distribution sites tended to be more restricted during the onset of the pandemic, with reduced capacity, shorter hours of operation, and the closure of some sites. One participant noted that “*the lineups are longer. The hours of the places that’s open is not as long… It’s harder for us to get all the tools* [e.g. harm reduction supplies] *we need*” (#27, medium urban). As this participant clearly shares, disrupted services had negative impacts on the ability of some to practice harm reduction and enact safer use practices recommended by public health agencies.

The range of services and resources offered at some shelters were reduced due to COVID-19 restrictions: “*You can come in* [to shelters], *but, like, you can’t hang around. They’re not giving out resources anymore. They’re not giving out food anymore. They’re not giving out clothes anymore. They’re not giving out safe using supplies*” (#36, large urban).

Participants from some rural communities in BC generally indicated more limited access to harm reduction and other essential support services for people who use substances in their communities both before and during the COVID-19 pandemic when compared to more urban communities.

Despite restrictions and adjustments to follow COVID-19 guidance, however, most individuals felt that they could still access the supplies that they required and no participant identified concerns about access to take-home naloxone. In fact, participants from some jurisdictions found that access to services was expanded in response to the pandemic, especially pipe distribution and streamlined access to opioid agonist therapy (OAT).

[COVID] *made getting me on methadone and stuff easier… I didn’t know before COVID you could just go… I can walk into the OAT clinic and talk to them, see a doctor that day and get a prescription that day. But then I seen like a poster thing about it and how quick it was or whatever after COVID. And, yeah, so – that made me want to go do it ‘cause it was quick and easy.* (#24, rural)

Participants noticed some reduction in barriers to OAT access, including in rural communities, with the introduction of the Risk Mitigation Guidance in 2020 to provide clinical direction to health care providers to support people who use drugs and prevent community spread of COVID-19 [35].

Participants spoke highly of local harm reduction organizations and the role of peer workers in counteracting reduced access to services due to COVID-19 guidelines through creative and persistent provision of harm reduction services throughout the pandemic. One participant explained: “*I have had no problems getting harm reduction supplies…* [the harm reduction supply site] *has been really supportive. They’ve been really helpful and not judgmental… They’re really cool*” (#6, large urban). Several participants described the temporary harm reduction supply sites that they operated out of their homes, garages, and vehicles to meet the needs of their communities when supplies were difficult to access: *“it was just me and my wagon because we didn’t have a vehicle. So I was walking around with all of the supplies for about six months.”* (#48, small urban).

When access was limited, some participants explained that they would request additional harm reduction supplies when accessing services to be able to distribute to their friends.

*Whenever I go* [to a harm reduction supply site] *I try and grab extra too because like I said, I know other people who use whatever. So I try and grab extra, so if anybody needs anything, whatever, if I have extra, I will help out with whatever I have. Like, I for instance have extra pipes or extra fucking brillo or a crack pipe or whatever, I will help other people out. Because I just feel like it’s necessary. Like, we’ve got to look out for one another. And nobody else is going to look out for us. So why not look out for one another, right?* (#6, large urban)

The commitment of participants to ensuring the safety of their peers and community members was a critical factor serving to protect against overdose, especially in the context of the pandemic.

#### Overdose response and additional burden on peer workers

Participants explained that the burden of emergency overdose response has historically been and continues to be placed on peer workers who are on the frontlines of the illicit drug toxicity crisis. In the COVID-19 context, participants pointed to rapidly evolving protocols and the introduction of additional safety precautions as a source of increased stress and confusion among peer workers: “*We’re loaded with all of this information as frontline workers, right, about extra precautions to take.*” (#49, small urban) and *“it’s a constantly changing thing as we learn more and… the restrictions are pretty fluid… constantly changing.”* (#44, metropolitan).

Particularly early in the pandemic, participants were concerned about how to give breaths during an overdose in a safe manner, especially when no face shield or protection is available. Peers and frontline workers also expressed frustration when they felt that emergency responders delayed attending to a person as they donned protective equipment after arriving at the scene of an overdose, due to COVID-19 precautions: “*I get frustrated dealing with* [emergency responders] *because I’m in distress myself. And I have to make sure that the person’s okay and it kind of seems to take forever* [for emergency responders to step in]” (#37, metropolitan).

*If an ambulance does attend – sure they’re masking and they’re gowning, they’re waiting… They’re not approaching a person in a rush situation… it’s become more relaxed which for frontline workers can cause a lot of stress and anxiety ‘cause here we are, we worked on this person and then we’re supposed to step away when a medical professional comes in. But we’ve got to wait for the gloves and the PPE* [personal protective equipment] *and so I think that* [overdose response] *has been affected for sure.* (#49, small urban)

Some participants also indicated that they were more hesitant to call 911 in the event of an overdose to prevent going to hospital and to minimize interaction with emergency responders and hospital workers out of fear of contracting COVID-19.

*I’ve noticed a lot of people if they were hanging out with a friend… and the friend went down, they would Narcan them and make sure they’re okay, but they would not call the ambulance when normally they would have. Because I think that’s because they don’t want to cross contaminate with people and stuff… they just don’t want to have to deal with the whole thing of going to the hospital and, you know, like dealing with the COVID situation going on in there… A lot of people have health issues when they’re using so going to the hospital is like, you know, the chance of getting COVID or getting a cold or something is pretty great and getting even sicker, you know.* (#46, large urban)

Despite the uncertainty surrounding safety and burden of overdose response in the pandemic context, participants highlighted the immense resiliency of their communities and their commitment to saving lives and acting to protect one another from harm. Participants described the ways that they and their peers have adapted throughout the pandemic by increasing outreach, establishing temporary services, and taking additional precautions to keep one another safe. Many participants spoke of taking additional safety precautions to protect themselves and their community from contracting the virus, including: wearing masks, asking questions about symptoms and potential contacts, and prioritizing use of new injection or smoking equipment more than pre-pandemic: “[going] *out of your way more to get a clean* [needle or pipe] *more so than you would have before* [the COVID-19 pandemic]” (#12, small urban).

The majority of participants also asserted that responding quickly and appropriately to overdoses took precedence over any concerns about contracting COVID-19:

*I don’t even have* [risk of COVID] *in consideration when I’m responding. It’s the least thing in my mind when I go to respond to an overdose. Maybe that’s naïve on my behalf, but – yeah, the person at risk is the first thing I’m concerned about.* (#32, large urban)

Although many of the factors described above were identified as increasing overdose risk and substance use-related harms in the context of the COVID-19 pandemic, participants also highlighted factors that protected against overdose and substance-related harm. These protective factors emerged through public health programs and the actions of communities of people who use drugs. This included: new programs, such as pipe distribution; expansion of peer outreach; the resilience of established social relationships; and individuals who consistently prioritized overdose response over concerns about COVID-19 transmission to care for one another.

Factors increasing and reducing overdose risk and substance use-related harms described by participants were nuanced and varied. The ‘risk environment’ framework examines factors affecting risk by way of physical, economic, social, and policy domains. Table 2 summarizes the thematic findings presented above using this framework for a complementary conceptualization of the key findings.

**Table 2:** Factors identified by participants as shaping overdose risk and substance use-related harms in the context of the COVID-19 pandemic, organized by adapting and applying the ‘risk environment’ framework [20, 21]

**Risk Environment Domain:** Physical	
**Factors Increasing Risk**	**Protective Factors**
- Physical distancing restrictions and isolation leading to mental health challenges and increased drug use - More use of substances alone without bystanders able to respond in the event of an emergency - Rural challenges (e.g. limited access to affordable and safe substances, fewer services for people who use substances) - Service restrictions and closures	- Increased outreach and temporary supply distribution sites led by people with lived and living experience of substance use
**Risk Environment Domain**: Economic	
**Factors Increasing Risk**	**Protective Factors**
- Spike in drug prices early in the pandemic - Loss of readily available economic opportunities - Inconsistencies in the drug supply	- Economic means to respond to rising drug prices
**Risk Environment Domain**: Social	
**Factors Increasing Risk**	**Protective Factors**
- Stigma towards people who use substances alongside perceptions of people who use substances as “infectious” - Isolation and increased use of substances alone, without bystanders able to respond in the event of an overdose - Burden of overdose response on peer workers	- Expanded outreach led by people with lived and living experience of substance use and local harm reduction organizations - Social drug use norms and social capital (e.g. trusted relationship with dealer, sharing supplies and substances, buddying up)
**Risk Environment Domain**: Policy	
**Factors Increasing Risk**	**Protective Factors**
- Public health orders, including physical distancing, that led to service restrictions (e.g. reduced hours of operation, visitor restrictions, and service closures) - Shifting COVID-19 protocols and increased burden of overdose response of peer workers - Delays in emergency responders stepping in when arriving at the scene of an overdose and personal protective equipment requirements - Border closures and supply chain disruptions	- New programs and policies emerging in the dual public health emergency (e.g. increased availability of pipe supplies, risk mitigation guidance)

## Discussion

This paper describes the ways that the COVID-19 pandemic and resultant public health responses altered the environments in which people live and use substances, thereby influencing risks of harm, including overdose. The pandemic has exposed the precariousness of the systems in place that are intended to protect the health of people who use substances [6]. Our findings highlight the ways in which policies put in place in the context of public health emergencies can disproportionately impact certain communities, including people who use illicit substances [36–38].

Participants pointed to physical distancing measures as being responsible for creating social and physical isolation and increased substance use. Often this led to people using alone in the absence of someone who could act as a bystander and provide an emergency response or call 911 in the event of an overdose. These patterns of use are particularly concerning given that most illicit drug toxicity deaths in BC occur in private residences or while using alone [39].

Consistent with available literature [14, 24, 25], drug prices spiked early in the pandemic and supply chain issues created inconsistencies in drug availability and more frequent “dry spells”, leading some individuals to change their drug of choice to match what was available. As described by our participants and the BC Coroners Service, substances available on the street are becoming increasingly toxic and dangerous due to impurities and contaminants, such as benzodiazepines and etizolam [40, 41]. Though participants explained the ways that they try to use substances safely, the context of the pandemic, combined with the drug toxicity, brought about an unpredictability that increased risk of overdose and death.

Amid the pandemic, other studies noted similar restrictions and reduced access to harm reduction services to those described by participants in this study [14, 25, 27, 42, 43]. These findings mirror the marked decline in the number of visits to supervised consumption services and overdose prevention sites in BC reported by the BC Centre for Disease Control during this time [16]. Despite these limitations, some participants felt that certain services improved during the pandemic, such as the roll-out of pipe distribution during this time period in addition to the introduction of BC’s risk mitigation guidance which seek to increase ease of access to pharmaceutical alternatives to illicit substances [44].

People who use substances continue to face stigma and discrimination both within the community and when accessing health services [45]. This has important implications on an individual’s ability to use substances in a safer manner, given that it has been well documented that stigma contributes to higher rates of people hiding drug use, avoiding accessing services, and using drugs alone [39]. Several participants described their personal experiences of stigma and discrimination and felt that stigmatization was accentuated by concerns of COVID-19 transmission [46], particularly due to public perceptions of people who use substances and the heightened ‘othering’ that resulted from the implementation of physical distancing measures [36].

Despite some participants expressing concerns about contracting COVID-19 when responding to overdoses, most participants shared that this would not change their overdose response approach because they prioritized saving lives over contracting the virus. These findings are aligned with those of Galarneau et al. (2021) who outlined similar priorities among participants who, similarly, described feeling proud and supportive of their community’s willingness to respond to overdoses and protect their community amidst a pandemic and associated risks that could conceivably deter people from responding to overdoses [25].

People who use substances have a long history of leading the way in developing strategies and establishing services to meet the needs of their communities when sanctioned services are not available (e.g. [47–49]). The resiliency, leadership, and commitment of people who use substances to equity and the wellness of their community as well as the creative actions taken against two public health emergencies were critical in shaping the risk environment for people who use substances. Participants reported ways community members adapted to protect individuals from harms due to the unregulated drug supply and the criminalization of drugs by operating makeshift harm reduction distribution programs and sharing substances when needed. However, participants described the extra burden placed on peers and frontline workers responding to overdoses and navigating through the constantly shifting COVID-19 guidelines that did not always reflect the concerns and risks faced by people who use illicit substances. Peer workers have been shown in the literature to be increasingly overburdened by the responsibility of responding to overdose and compensating for program, policy, and funding deficiencies, which in turn contributes to experiences of emotional stress and burnout [50–52]. The COVID-19 pandemic has exacerbated existing stressors and burnout for peer workers and other staff working harm reduction services [53]. These findings call for greater support for peer workers who bear the burden of responding to overdoses and providing care for people who use substances [47, 52, 54], while often receiving minimal recognition and resources [52].

Our findings underscore the importance of preparing for future public health emergencies or disruptions to services, such as future pandemics or natural disasters [25], and ensuring that the needs of people who use substances are prioritized. It is imperative that people with lived and living experience of substance use are engaged at every stage of the development and implementation of social and public health policy [55], especially during times of emergency. The continuum of services available must be timely, responsive, consistent, and tailored to meet the diversity of needs and preferences of people who use substances [27]. These responses should account for the complex physical, social, economic, and political environments that shape drug use and risk of harms. Harm reduction services must remain open, operational, and scaled up in times of emergency [27], alongside additional low-barrier approaches, such as spotting [47] or other remote methods of supervision and overdose response [56–58]. Our findings are relevant to post-pandemic circumstances given the high likelihood of future service disruptions and displacement of people who use substances in the context of major climate or environmental disasters, such as wildfires and flooding, which are occurring at increasing rates throughout BC [59].

Finally, our findings speak to the precarious nature of the unregulated drug supply exacerbated by the COVID-19 pandemic. As such, expanded implementation of a legal, regulated supply of substances would help to combat the toxicity of the existing unregulated drug supply and to address the “dry spells” and inconsistencies in supply described by participants that caused them to switch to other substances and turn to unknown suppliers. BC introduced the Risk Mitigation Guidance in the context of the COVID-19 pandemic which has brought benefit for some people who use substances in reducing reliance on illicit substance use and overdose risk, but requires further optimization to adequately meet the diversity of needs of individuals accessing the program [60]. Pursuing decriminalization would also help to address the stigma described by participants, reduce the harms of criminalization, and minimize barriers faced by people who use substances when accessing health, social, and harm reduction services [14, 47]. Several jurisdictions have taken steps to decriminalize possession of some illicit substances for personal use within their communities, including Vancouver, Toronto, and most recently the Province of BC where legislation came into effect on January 31, 2023 [61]. Calls for decriminalization and expanded roll-out of safer supply have intensified during the COVID-19 pandemic given the urgency of finding solutions to address these dual public health crises and to protect the health and rights of people who use substances [62].

This study has several limitations. First, interviews were conducted between the months of October 2020 and May 2021, and therefore represent the perspectives and experiences of those interviewed during this period only. Given the ongoing, shifting context of the COVID-19 pandemic and evolving public health restrictions, the findings may not be representative of experiences later in the pandemic. Similarly, interviews were conducted in the early stages of vaccine roll-out; as such, we were not able to explore the implications of vaccine availability on risk perceptions among participants. Although participants in this study were diverse with respect to age, gender identity, urbanicity, and indigeneity, this study was conducted in BC, therefore, results may not be generalizable to other Canadian jurisdictions. We did not specifically ask which substances each participant was currently using other than the broad categories of opioids and stimulants, therefore, we were unable to fully characterize or contextualize overdose risk or which substances participants switched to in the context of a shifting drug supply. Most interviews were conducted over the phone and therefore demanded additional planning and access to a phone. This may have excluded certain individuals, though PRAs and interviewers were often able to assist by providing a phone for the interview or by arranging to meet in-person to facilitate involvement. Despite many interviewers being PRAs and all being experienced and skilled at creating a safe, comfortable environment for participants to share their experiences, data may still be affected by social desirability bias, especially when discussing substance use behaviours and adherence to public health guidelines amid the COVID-19 pandemic. A major strength of our study is the large sample size (n = 62), which enabled saturation was reached on the consequences of COVID-19 on overdose risk. However, more research is needed to explore the lingering impacts of COVID-19 on people who use substances.

## Conclusion

This study illustrates the complex contextual factors that shape overdose risk and the ways that the pandemic has brought to light the ongoing gaps in services for people who use substances. Policies put in place in the context of public health emergencies disproportionately impacted certain communities, such as people who use substances, and the findings highlight the importance of ensuring that the needs of people who use substances are addressed and prioritized in future emergency responses. Moving forward, the continuum of services must be flexible, adaptable, and tailored to meet the diversity of needs and preferences of people who use substances. This should also include low-barrier approaches that are able to be scaled-up in times of increased demand. Decriminalization and progressing towards a legal, regulated supply of substances is essential to address the increasingly toxic drug supply and to minimize the stigma and barriers related to accessing health and social services.

## Additional notes

We want to acknowledge that “*It’s just a perfect storm*”, as used in the title of this paper, is a quote from participant #53. Though highlighting the perspectives of one participant does not adequately communicate the nuances of the topics that are expanded upon in the body of the paper, we felt that this quote was a broad enough to encapsulate the views of the majority of participants regarding how the COVID-19 pandemic has affected overdose risk in BC. Inclusion of this quote in the title of this paper was also supported by our peer advisors. We acknowledge the shortcomings of such an approach, as outlined by Parkin and Kimergard [63].

## Data Availability

The qualitative datasets for this study are not publicly available given the sensitive nature of the topic, including confidential information that could compromise participant confidentiality and consent. The data analyzed during the current study are available from the corresponding author on reasonable request.

## List of abbreviations

BC: British Columbia
COVID-19: Coronavirus Disease 2019
CUT Meth OD: Concurrent Use and Transition to Methamphetamine Among People at Risk of Overdose
GSDOA: Good Samaritan Drug Overdose Act
OAT: Opioid Agonist Therapy
PEEP: Professionals for Ethical Engagement of Peers
PRAs: Peer Research Assistants.
